# Ankle Injuries in Soccer Players: A Narrative Review

**DOI:** 10.7759/cureus.17228

**Published:** 2021-08-16

**Authors:** Spyridon Kolokotsios, Gianna Drousia, Ioannis Koukoulithras, Minas Plexousakis

**Affiliations:** 1 Department of Physical Therapy, University Hospital, University of West Attica, Athens, GRC; 2 Department of Orthopaedic Surgery, Faculty of Medicine, University of Loannina, Athens, GRC

**Keywords:** injury epidemiology, soccer, ankle injuries, soccer player, football

## Abstract

Soccer is one of the most popular sport, with many describing it as the "king of sports." In recent years, increased global participation in soccer has led to an inevitable increase in injury rates, especially in the lower extremities. Consequently, there is an increase in the epidemiology of soccer injuries, both in professionals and amateur athletes. The cause of an injury is multifactorial and depends on psychosocial, predisposing, intrinsic, and extrinsic factors. Also, contact with another player and non-contact injuries seem to be the most widespread mechanisms of injuries. The most common injuries recorded in soccer are ankle sprains and hamstrings injuries. More specifically, many studies have shown a correlation between the previous injury in lower extremities, weakness of abductors muscle, and psychosocial factors with the ankle sprain. Additionally, according to study results, injuries in adult men, adolescent men, and women during a match are higher than injuries during training. This narrative review aims to record the epidemiology of ankle injuries, risk factors, and the relationship between circadian rhythm, sleep, and injuries.

## Introduction and background

According to the United States National Sports Injury Registration System, a soccer injury is defined as any related accident that occurs during training or a match. The consequence of this is the restriction of the player’s participation for at least one or more days, depending on the severity of his condition (Table 1) [1]. Re-injury is defined as a type of injury such as a fracture or ankle sprain that occurs in the same anatomical area that the athlete has previously suffered [2]. A player is considered injured after being diagnosed by the team doctor until he returns to his duties and consequently on the field [2]. Based on the days of abstinence from athletic activity, musculoskeletal injuries can be classified into severity levels (Table 1). Soccer's unique injury profile is categorized as acute (e.g., sprains, muscle fractures, tendon injuries, dislocations, fractures) and chronic, based on their etiology [3].

**Table 1:** Classification of injuries by severity level and days of abstinence.

Severity levels	Days of abstinence
		Slight/minimal	0-3 days
Mild	4-7 days
Moderate	8-28 days
Severe	28+ days

## Review

Epidemiology of soccer injuries

Epidemiological studies of soccer injuries have been conducted in Europe since the late 1970s. Over the last three decades, soccer participation has increased, and consequently, more injuries have been recorded during training or matches compared to previous decades [2]. The cause of injuries depends on several factors. In particular, it has been reported that long periods lead to fatigue in athletes, resulting in an increased risk of ankle joint injuries [4]. Additionally, the injury factors include frequent acceleration and deceleration of players, abrupt changes of direction, and repetitive kicks [4].

The frequency occurrence of soccer injuries during matches is, on average, four to six times higher than the incidence of injuries that occur during training [1]. A relevant study of former soccer athletes found a higher predisposition to developing osteoarthritis of the ankle (6%) in former professionals than in the general population. Thus, it is necessary to identify the causes and mechanisms of injuries in the area of ​​the foot [1].

In recent years, artificial turf surfaces have been widely used due to their low maintenance costs and increased usability in various environmental conditions. Indeed, the Federation Internationale de Football Association (FIFA) has approved the new generation artificial turf for soccer matches at the elite level of athletes. However, most soccer players still prefer natural grass. This is directly related to the significantly increased number of injuries, discomfort, and fatigue athletes feel on the artificial turf [5].

In soccer, ankle sprains and muscle injuries are the most common types of injuries. More specifically, there is a higher percentage of muscle strains in the hamstrings, quadriceps, hip, and gastrocnemius. Moreover, according to Van Dijk et al., most muscle injuries occur in the first half time between 30 and 45 minutes of the match [2].

**Figure 1:**
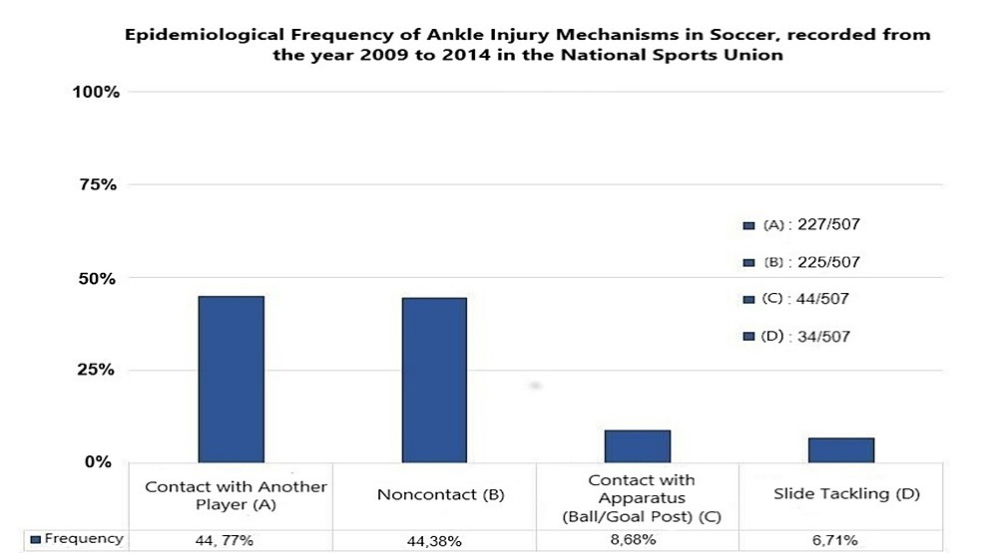
The epidemiological frequency of the injury mechanism, recorded from the year 2009 to 2014 in the NSU. NSU: National Sports Union.

The most common mechanisms of injury in the ankle area are contact with another player, noncontact injury, contact with apparatus (ball/goal post), and while being slide tackled [6]. Gulbrandsen et al. recorded the frequency of ankle injuries mechanism during training and games [6]. Figure 1 displays the epidemiological frequency of the injury mechanism, recorded from the year 2009 to 2014 in the National Sports Union (NSU).

In soccer, the ankle joint is one of the most common anatomical injury areas. The most common injuries are the external and internal lateral ligaments (external and internal sprain), injuries of blood vessels, high ankle sprains, and myotendinous injuries. Despite the multiple pathologies affecting the ankle joint, approximately 80 ± 10% of injuries are diagnosed as sprains [2].

In a ten-year epidemiological study, ankle injuries were investigated during the match and training periods. Between 2004 and 2009, 66.82% of all injuries were on the external lateral ligament (external ankle sprain). Meanwhile, 8.71% of all injuries were on the internal lateral ligament (internal ankle sprain), 9.48% were injuries of blood vessels, and 11.47% were high ankle sprains. However, this study showed that between 2009 and 2014, injuries on the external lateral ligament (external ankle sprain) were reduced to 62.13%, while injuries on the internal lateral ligament were accreted to 13.82% (internal ankle sprain). At the same time, 11.83% of all injuries were injuries of blood vessels, and 6.51% were high ankle sprains [6].

Walden et al. studied all injuries in the ankle area without being limited to recording ankle sprains. This study concluded that the incidence of sprains has been declining since 2001, suggesting the effectiveness of injury prevention programs [7]. Nevertheless, ankle sprains are still among the most widespread injuries in soccer traumatology [7].

Epidemiology of injuries in women's football

In recent years, there has been an equally rapid growth in the sport of women's soccer. The incidence of injuries for women ranges between 1.2 and 7 injuries per 1,000 hours of training and 12.6 - 24 per 1,000 hours of the match. Therefore, the soccer match carries a significantly higher risk of injury than the training of female athletes [8].

Injuries in women's soccer are mainly observed in the ankle joint and the knee joint [9]. At the same time, there is a high epidemiological rate of injuries in hamstrings and gastrocnemius muscles. In contrast, hip and groin injuries are less common in women than in male soccer players [8]. Also, ankle joint injury rates have been similar between male and female professional athletes during the football season [8].

Epidemiology of injuries in adolescent football

Adolescent soccer players are exposed to intense training and constantly participate in football matches. At the same time, the age that athletes go through is characterized by periods of rapid growth and maturation of collagen tissue. Therefore, during adolescence, excessive strain on athletes contributes to increased injury rates in various anatomical areas of the body [10].

According to a recent cohort study by Wik et al., the frequency of injuries in matches was 3.9 times higher compared to the frequency of injuries in training (32 and 8.2 per 1000 hours, respectively). At the same time, the most common anatomical areas of injury were recorded in adolescent soccer players. Specifically, the lower extremities show the highest epidemiological incidence of injury (83%), followed by the upper extremities (9%), the trunk (6%), and the head - the neck (2%) [10].

The percentage of teenage players who had at least one injury during a season was 78.5%. Respectively, the percentage of players who had a re-injury was 12%. Therefore, the overall occupancy of athletes decreased to 85.1% for training and 89.6% for matches [10].

Injuries to the hamstrings muscles and ankle sprains have a high epidemiological frequency, reflecting the intense nature of the sport [10]. The highest epidemiological frequency occurs in muscle injuries - fractures (three injuries per 1000 hours). However, the effects of sprains on the ankle joint are more significant than muscle injuries due to the higher abstinence in days from the sport [10]. 

Epidemiology of injury over time

The ultimate goal of soccer clubs is to win matches. Therefore, avoiding injuries plays a key role in the success of the team. The low injury rate allows most members of the team to participate in training and matches actively. In fact, according to recent studies, injuries during the match and training decrease over time while at the same time increasing the availability of players [11].

More specifically, an 18-year cohort study recorded approximately 12,000 injuries in 1.8 million hours of play. The results showed a significant reduction in the frequency of injuries by 3% per season (training and matches). Ligament injuries decreased by 5% per training season and by 4% per match season. However, no significant reduction in muscle injury was seen over time [11].

In contrast, serious ligament injuries appear to have increased by 4% per training season and by 1% per match season. The overall severity of injuries decreased by 2% per match season, and there was no statistically significant difference throughout training. In addition, the possibility of re-injury appears to have decreased by 5% per match and training season. In particular, there was a reduction of ligament injury during training and matches by about 7%. On the other hand, muscle injuries showed a reduction during training but not during the match [11]. Finally, the overall reduction in athletes' injuries has resulted in increased staffing of soccer clubs. Therefore, the availability of players both in training and match increased by 0.7% and 0.2%, respectively [11].

Predisposing factors of foot injury

Soccer involves high-intensity activities, which over time can cause musculoskeletal injuries to players. The incidence of injuries in soccer ranges from 7.4 to 47.5 per 1000 hours of play. This significant variation is due to various predisposing factors, such as the different intensity of training, the age of the players, the gender, and the research design differences. The majority of injuries in soccer (68-88%) occur mainly in the lower extremities and, more specifically, in the ankle and hamstrings [12].

The ankle joint absorbs the mechanical loads produced by the interaction of players with the ground. This makes the joint prone to injuries, such as sprains, which affect the lateral ligaments, and the disability depends on the degree of injury. The repetitive sprains can lead to mechanical instability of the joint [13].

Injuries are widespread in soccer. Due to the high frequency of injuries, it was necessary to conduct studies related to identifying and recording predisposing factors. However, it seems that predisposing factors are not directly related to the onset of injuries, and for this reason, there have not been conducted many studies. Soccer players who exhibit a muscular imbalance between the femoral biceps and quadriceps with previous injuries of femoral biceps appear to be more prone to femoral biceps re-injury [14].

A study conducted by Powers et al. studied the association of hip abductor weakness with an ankle sprain. The findings showed that the chances of causing a sprain from non-contact injury increase by ⋍10% for each unit of force that decreases, expressed as % body weight (BW). More specifically, when the strength of the hip muscles is ≤33.8% BW (player with a high risk of injury), the risk of injury increases from 11.9% to 26.7% [15]. In a similar study, it was studied the correlation between hip extensor weakness and ankle sprain. According to the study results, there is no correlation [16].

Psychosocial risk factors

In sports, mental health is two-way, as on the one hand, engaging in sports activities is likely to prevent mental disorders, while on the other hand, stress contributes to the development of possible depression or anxiety [17].

Slimani et al. showed that psychosocial factors might be directly related to increased rates of injuries in soccer players. More specifically, personality traits (such as stress and perception) and exposure to stressor factors (high level of daily stress, daily fatigue, and previous injuries) are directly related to most injuries [18].

Sleep is very important for the athlete's recovery after an injury. Poor sleep is observed mainly before the match season and may significantly affect athletes' performance and skills [19]. Leproult and Van Cauter observed a reduction in testosterone levels between 10% and 15% within a week in healthy individuals who slept five hours a day, thus proving that lack of sleep can adversely affect the musculoskeletal system, and musculoskeletal injuries can occur. More specifically, during sleep, a release of anabolic hormones (testosterone, cortisol) has been observed. These hormones contribute to the synthesis and breakdown of muscles proteins, so they help restore muscle after exercise. Sleep disorders are associated with reducing these hormones levels, so it appears that muscle integrity and growth are affected [20].

Silva et al. recorded the injuries about the quality of sleep in an elite team of footballers for six months. Their findings showed that the quality and duration of sleep were associated with musculoskeletal injuries. More specifically, in a total of 28 players with muscle injuries, 27 of them had poor quality of sleep and 1 good quality of sleep [19]. Table 2 synopsizes the relationship between the predisposing factors with hamstrings and ankle injuries.

**Table 2:** The relationship between the predisposing factors with hamstrings and ankle injuries.

Predisposing factors for injuries	Hamstring injury	Ankle sprain
The muscular imbalance between the hamstrings and the quadriceps	Correlation observed	No correlation observed
Previous injury	Correlation observed	Correlation observed
Reduced range of motion of hamstrings	No correlation observed	No correlation observed
Decrease dorsiflexion of the foot	No correlation observed	No correlation observed
Weakness of hip abductor muscles	No correlation observed	Correlation observed
Weakness of hip extensor muscles	No correlation observed	No correlation observed
Psychosocial factors	Correlation observed	Correlation observed

Intrinsic and extrinsic risk factors

The factors that contribute to injuries are divided into intrinsic and extrinsic. Intrinsic risk factors include players' anatomical features, such as increased leg width, functional isokinetic deficits, and kinesthesia. In addition, intrinsic factors have been associated with an athlete's age, a possible previous injury, and an increased body mass index [12]. One of the theories regarding the etiology of sports injuries is the stress-strain capacity model. This model is mainly focused on the athletic behavior of the players, as the athlete is no longer considered a passive recipient of stress but as his active manager. Consequently, sports injuries result from a complex interaction of various factors that contribute to the provocation of injuries [21].

**Figure 2:**
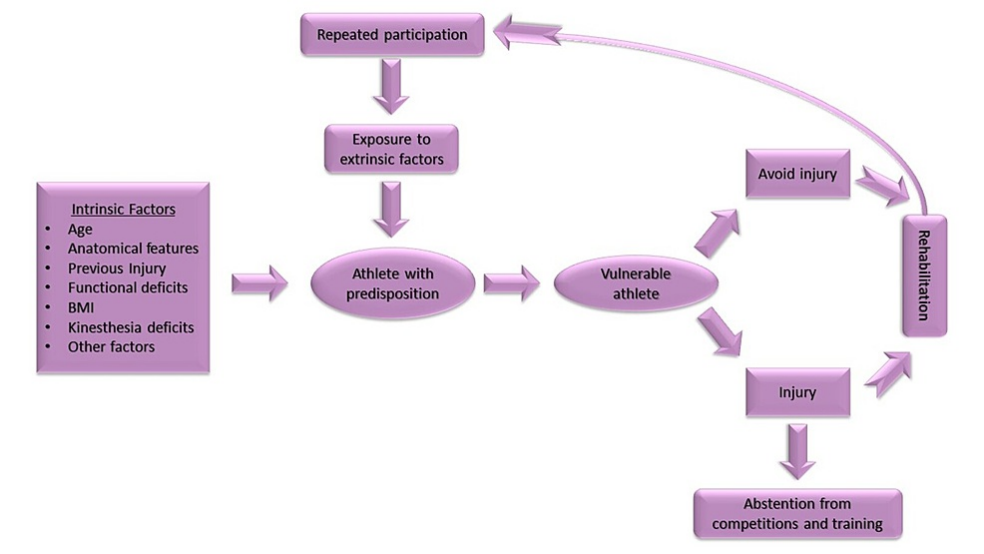
Illustration of the interaction of intrinsic and extrinsic risk factors in athletes. BMI: body mass index.

On the other hand, extrinsic factors seem to be related to the type of sports activity, exercise, environmental conditions, and equipment used [21]. In addition, insufficient warm-up, direct contact by the opponent, and the use of artificial turf are extrinsic risk factors [12]. Figure 2 presents the interaction between the intrinsic and extrinsic risk factors in athletes.

Circadian rhythm and injuries

Circadian rhythm is a term used to describe various human factors that may affect athletic performance in relation to the time of day. It has been argued that fine motor skills increase in the morning, while gross mobility usually shows maximum performance early in the evening [22].

Usually, optimal athletic performance is associated with increased body temperature and norepinephrine production. Therefore, if exercise is not performed during the day when the above biological changes are observed, it is likely to cause physical and mental fatigue, potentially increasing the risk of injury [22].

The aim of the study by Brogden et al. [22] is to investigate the effect of circadian rhythm, through clinical and functional exercises, on male semi-professional soccer players, with an ankle sprain. The findings of this study showed that circadian rhythm did not affect the performance of proprioception, isokinetic strength, and stability in the form of the star excursion balance test (SEBT). However, through an unstable platform biodex stability system (BSS), the stability assessment shows a significant effect of the circadian rhythm, which was observed at 12:00 noon and 19:00 pm.

Therefore, it is necessary to conduct new research on the possible association of the circadian rhythm with the prevention and rehabilitation of sprains in athletes.

Contemporary guidelines for treating soft tissue injuries

Muscle injuries are one of the most common traumas which occur in the sports field [23]. The clinical protocol of PRICE (protection, rest, ice, compression, and elevation) was the first-line treatment in acute soft tissue injuries for many years. Nowadays, there is controversy from many researchers about the effectiveness of this protocol [24].

In 2012, the PRICE protocol was replaced from the POLICE (protection, optimal loading, ice compression, and elevation) by Bleakley, Glasgow, and MacAuley. They proposed that rest should be replaced from optimal loading, while the early activity encourages early recovery [24].

In early 2019, Blaise Dubois and Jean-Francois Esculier proposed PEACE (protection, elevation, avoid anti-inflammatory drugs, compression, education) and LOVE (load, optimism, vascularization, and exercise) [25]. Unexpectedly, this clinical protocol revokes cryotherapy despite the widespread usage among clinicians and non-clinicians.

Miranda et al., in their systematic review, investigate the effectiveness of cryotherapy on pain intensity, swelling, range of motion, function, and recurrence in people with an acute ankle sprain. Their findings show that cryotherapy does not enhance effects of other interventions on swelling (MD ¼ 6.0; 95%CI: 0.5 to 12.5), pain intensity (MD ¼ 0.03; 95%CI: 0.34 to 0.28) and range of motion (p > 0.05) [26]. In fact, there is no high-quality evidence to support the efficacy of ice for treating soft tissue injuries [27]. Despite the short analgesic effect that may produce, cryotherapy could disrupt the inflammation process, angiogenesis, and revascularization. Moreover, cryotherapy could delay neutrophil and macrophage infiltration as well as increase immature myofibers [28]. Consequently, it may lead to impaired tissue repair and redundant collagen synthesis [28].

Muscle tears occur when the force applied to the tissue is higher than the tissue can withstand. Thus, muscle fibers get damaged in the light of rapid eccentric contraction, usually during acceleration or deceleration movements [29]. At the acute phase (degeneration/Inflammation), the complete rest, ice, and non-steroid anti-inflammatory drugs (NSAIDs) should be avoided according to PEACE and LOVE protocol. At the subacute phase (regeneration phase), mobilization and progressive loading are recommended to help the healing process and reduce scar tissue [30]. In the third phase (remodeling phase), a dynamic stretching program is recommended over static stretching in order to prevent the loss of eccentric force of hamstring muscles and functional performance [31].

## Conclusions

Soccer is a highly dynamic sport with high energy requirements and multiple fitness parameters. High levels of fitness are prerequisites for performing the skills of the sport, where the lower extremities are mainly involved. The global increase in participation in soccer, both professional and amateur, inevitably leads to an increase in the rate of injuries. From the analysis of the present study results, it is found that the cause of an injury depends on a number of psychosocial, predisposing, intrinsic, and extrinsic factors. Injuries occur in the contractile structures (muscles) and non-contractile structures, such as ligaments, bones, blood vessels, and skin. According to the present study's findings, the foot is one of the most common anatomical areas, which suffers the most injuries. Specifically, the incidence of injury of the external lateral ligament is 62.13%, while the incidence of injury of the internal lateral ligament is 13.02%.

From the results of the studies, there was a more significant increase in injuries in the 30th to 35th minute of the soccer match. Especially for the sprain in the ankle joint, the incidence of injury in men is 75.7%, while in women, 51%. The most common mechanism of sprain injury is direct boredom from an opponent. The incidence of injuries in adult men during a match is four to six times higher than the incidence of injuries during a workout. In adult women, the incidence of injury ranges between 1.2 and 7 injuries, per 1,000 hours of training and 12.6 and 24, per 1,000 hours of competition. Moreover, in adolescent men, the incidence of injuries in competitions is 3.9 times higher than injuries in training. Also, injury epidemiological studies found that ligament injuries were reduced by 5% during training and 4% during competition. However, serious ligament injuries have increased by 4% during training and 1% during competition. Last, but not least, the chances of re-injury seem to have decreased by 5%, both in training and in the season series.
